# The mutation rate of mycobacterial repetitive unit loci in strains of *M. tuberculosis* from cynomolgus macaque infection

**DOI:** 10.1186/1471-2164-14-145

**Published:** 2013-03-05

**Authors:** Mark N Ragheb, Christopher B Ford, Michael R Chase, Philana Ling Lin, JoAnne L Flynn, Sarah M Fortune

**Affiliations:** 1 Department of Immunology and Infectious Diseases, Harvard School of Public Health, Boston, MA, USA; 2 Department of Pediatrics, Children’s Hospital of Pittsburgh of the University of Pittsburgh Medical Center, Pittsburgh, PA, USA; 3 Department of Microbiology and Molecular Genetics, University of Pittsburgh School of Medicine, Pittsburgh, PA, USA; 4 Ragon Institute of MGH, MIT, and Harvard, Boston, MA, USA; 5 Broad Institute of MIT and Harvard, Cambridge, MA, USA

**Keywords:** *Mycobacterium tuberculosis*, Mycobacterial interspersed repetitive units, MIRU, Molecular epidemiology, Copy number variation, Whole-genome sequencing, Read depth, Paired-end mapping, Mutation rate

## Abstract

**Background:**

Mycobacterial interspersed repetitive units (MIRUs) are minisatellites within the *Mycobacterium tuberculosis* (*Mtb*) genome. Copy number variation (CNV) in MIRU loci is used for epidemiological typing, making the rate of variation important for tracking the transmission of *Mtb* strains. In this study, we developed and assessed a whole-genome sequencing (WGS) approach to detect MIRU CNV in *Mtb.* We applied this methodology to a panel of *Mtb* strains isolated from the macaque model of tuberculosis (TB), the animal model that best mimics human disease. From these data, we have estimated the rate of MIRU variation in the host environment, providing a benchmark rate for future epidemiologic work.

**Results:**

We assessed variation at the 24 MIRU loci used for typing in a set of *Mtb* strains isolated from infected cynomolgus macaques. We previously performed WGS of these strains and here have applied both read depth (RD) and paired-end mapping (PEM) metrics to identify putative copy number variants. To assess the relative power of these approaches, all MIRU loci were resequenced using Sanger sequencing. We detected two insertion/deletion events both of which could be identified as candidates by PEM criteria. With these data, we estimate a MIRU mutation rate of 2.70 × 10^-03^ (95% CI: 3.30 × 10^-04^- 9.80 × 10^-03^) per locus, per year.

**Conclusion:**

Our results represent the first experimental estimate of the MIRU mutation rate in *Mtb*. This rate is comparable to the highest previous estimates gathered from epidemiologic data and meta-analyses. Our findings allow for a more rigorous interpretation of data gathered from MIRU typing.

## Background

The ability to genetically differentiate among microbial strains facilitates tracing the origins and spread of bacterial pathogens, including *Mycobacterium tuberculosis* (*Mtb*), the causative agent of tuberculosis (TB). Various methods for genetically typing clinical strains of *Mtb* have been developed [1-4]. This includes typing strains through copy number variation (CNV) in mycobacterial interspersed repetitive units (MIRUs), which are minisatellite loci in the *Mtb* genome [5]. This approach, termed MIRU-VNTR (variable number of tandem repeats; hereafter, MIRU) typing, distinguishes genetically divergent strains rapidly and with relatively high accuracy [6]. As a result, MIRU analysis has been employed in a wide array of epidemiological studies [7-10], where an identical MIRU profile between isolates is interpreted as a recent transmission event [11]. This is based on the assumption that over short periods of time, a change in MIRU copy number is unlikely. Therefore, accurate estimation of the MIRU mutation rate is essential to infer the relationship between transmitted strains based on typing profile. The limits of this approach are highlighted by a recent study of a TB outbreak in British Columbia, where MIRU typing identified a single clonal outbreak, yet higher resolution WGS established two separate, simultaneous outbreaks [12].

Approximating the rate of MIRU CNV based on the profiles of clinical strains has proven to be a challenge. The approaches taken by various groups to estimate a rate have relied primarily on modeling of epidemiological data and meta-analyses [13-16]. The discrepancies in methodology, loci analyzed, and underlying assumptions between different studies have resulted in estimates ranging over two orders of magnitude. For example, Grant et al. (2008) analyzed copy number changes between *Mtb* lineages and used previous research estimating the time of the most recent common ancestor between lineages to estimate a MIRU mutation rate, yielding a per locus, per year rate of 1.05 × 10^-5^. Reyes and Tanaka (2010) used an infinite alleles model to define a relative rate and then benchmarked this rate against estimates of the IS6110 mutation rate to infer a MIRU mutation rate (7.00 × 10^-4^- 1.50 × 10^-2^). More recently, Aandahl et al. (2012) developed a stepwise mutation model for MIRU evolution and utilized Bayesian statistics to estimate the MIRU mutation rate of previously gathered epidemiological data (3.55 × 10^-3^), supporting the estimates provided by Reyes and Tanaka (2010).

Here, we seek to experimentally determine a mutation rate by assessing CNV in *Mtb* strains isolated from cynomolgus macaques [17], an animal model of *Mtb* infection that closely recapitulates the course of human disease [18]. The genomes from *Mtb* isolated from infected macaques were previously sequenced using the Illumina platform [17]. While protocols on detecting single nucleotide polymorphisms (SNPs) and small insertion/deletions (indels) using Illumina sequencing data are well established, it is less clear how to best determine the copy number in minisatellite loci. As WGS becomes increasingly common in epidemiological studies, the ability to establish MIRU copy number from sequencing data becomes important for the analysis of new WGS data in the context of previously existing typing data.

The length of Illumina reads fails to span the majority of MIRU repeats in a locus, which range from one to five or more repeats of 40–100 basepairs each in *Mtb*. Thus, short read sequencing cannot capture unique sequence and define copy number. Recently, researchers have utilized read depth (RD), a measure of the density of sequencing reads at each nucleotide in the genome, in order to identify CNV [19,20]. This approach relies on the observation that the absolute number of sequencing reads mapped to a reference genome is proportional to the copy number of a particular strain [19]. However, this approach has been primarily utilized to identify large (> 1 kb) variants in human tandem repeats, and RD has not been successfully applied to assess smaller minisatellite variation in microbes. Similarly, attempts to identify structural variation, including CNV, using mate pair distance have been previously employed in human genome studies [21-23]. This approach, termed paired-end mapping (PEM), utilizes the likely distance between paired reads from Illumina sequencing to identify structural variants. When mapping back to a reference genome, if the distance between paired reads is discordant from the expected value, it is suggestive of CNV relative to the reference genome. PEM has been successfully employed to identify large insertions or deletions. However, bacterial minisatellite CNV produces only small discordances relative to the reference genome which fall within the expected distribution of mate pair distance, making such events difficult to detect.

In this study, we sought to define a WGS methodology useful for identifying MIRU CNV by mapping sequencing reads to a single copy-number reference genome. We validated the WGS analyses by resequencing all MIRU loci via Sanger sequencing, which allowed us to assess the accuracy of using RD and PEM approaches to identify minisatellite variations. We then used our WGS and Sanger resequencing data to estimate a MIRU mutation rate during the course of infection. This rate will help guide the analysis of epidemiological data and provide a preliminary understanding of site-specific mutability in *Mtb*.

## Results

### Identifying MIRU CNV’s using WGS

We analyzed WGS data from 16 sequenced strains [17] for CNV at 22 of the 24 MIRU loci currently standardized for strain typing [6]. The remaining two loci showed poor read density and were excluded from this portion of the analysis. We utilized RD and PEM, both of which have been shown to correlate with copy number, to identify MIRU CNV. We hypothesized that any strain containing a MIRU insertion would exhibit an increase in RD and a decrease in mate pair distance relative to the input strain, while a deletion would exhibit decreased RD and increased mate pair distance [19,20]. However, either method is complicated by the inherent limitations of mapping short reads.

Illumina reads corresponding to MIRU regions often cannot be mapped unambiguously. This poses a challenge to identifying MIRU CNV, as it may reduce or alter signal at these loci. Different sequence alignment software packages have attempted to circumvent the challenge of assigning reads that map to multiple sites (termed multi-reads) [24-29]. Algorithms may discard multi-reads, place all of them at one potential mapping site, or randomly distribute them to multiple mapping sites (for a review of mapping multi-reads see [30]). We reasoned that directing multi-reads to a single repeat unit in the reference genome would result in more discrete and predictable mapping. Therefore, we sought to reduce ambiguity in mapping repetitive elements by constructing a reference *Mtb* H37Rv genome with only a single MIRU copy at each locus. This approach resulted in 16 loci with a single MIRU copy and 6 loci with two unique MIRU copies, which we subsequently treated as independent MIRU loci (A and B, where at least 5 SNPs distinguish A & B) for mapping purposes (Table 1). As expected, comparing the average mate pair distance for reads mapped to the H37Rv genome and the single copy-number genome reveals a significant difference in mapping at the MIRU site (p = 1.40 × 10^-04^; Table 2, Additional file 1: Figure 1a, 1b). Mapping differences were restricted to reads at the MIRU, as the average mate pair distance of reads flanking the MIRU region (+/− 100 bp) is not significantly different when comparing the two reference genomes.

**Table 1:** List of CNVs identified via WGS and Sanger resequencing

**MIRU locus**	**Read depth candidate strains**	**Mate pair candidate strains**	**Copy numbers**
154-A	None	None	1
154-B	None	None	2
424	None	None	2
577	None	None	2
580	A-3	None	3
802	**H-3**	**H-3**	3; (H-3 = 4)
960-A	I-2	None	3
960-B	None	None	1
1644	None	None	3
1955-A	None	None	1
1955-B	None	None	1
2059	None	None	1
2163b	None	None	3
2165*	None	None	3
2347	None	None	1
2401-A	None	None	3
2401-B	None	None	1
2461	None	None	2
2531-A	None	None	1
2531-B	None	None	1
2687-A	None	None	1
2687-B	None	None	1
2996	None	C-1	5
3007	None	None	3
3171*	None	None	2
3192	**G-3**	C-1	2; (G-3 = 1)
3690	None	C-1	5
4052	None	None	3
4156	None	None	3
4348	None	None	2

Loci containing asterisks (2165 and 3171) were only analyzed using Sanger sequencing. Bolded strains (H-3 at locus 802 and G-3 at locus 3192) were confirmed insertion and deletion events, respectively, via Sanger resequencing. Discrete copy numbers for each strain were determined by Sanger sequencing. Previous strain notation is used [17].

**Table 2:** Comparing mean mate pair distance between H37Rv and single copy reference genomes

**Strain**	**H37Rv (MIRU)**	**Single copy (MIRU)**	**H37Rv (+/− 100 bp)**	**Single copy (+/− 100 bp)**
A-1	165	128	157	146
A-3	188	137	178	165
B-1	163	124	153	142
C-1	156	120	148	138
C-2	206	159	190	174
E-1	205	159	188	173
F-1	174	161	172	169
G-1	157	119	147	137
G-3	235	168	203	177
H-2	201	159	188	174
H-3	199	155	184	170
I-1	218	180	208	193
I-2	230	187	218	200
I-3	223	183	204	198
I-4	224	176	212	193
I-6	204	157	184	171
I-7	202	151	182	166

Mean mate pair distance values were calculated for each strain at MIRU locus 3192. Values were gathered for the MIRU coordinates as well as the surrounding (+/100 bp) region.

**Figure 1:**
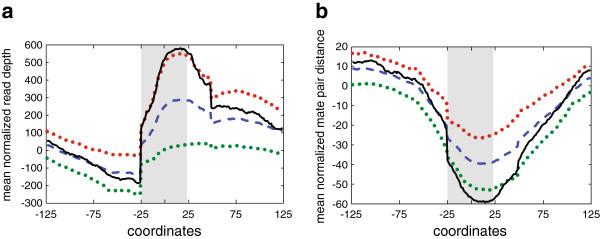
**Identifying MIRU CNV utilizing RD and mate pair.** (**a**) Mean normalized RD values at MIRU locus 802 (+/− 100 bp). Mean RD values represented by dashed blue line while two standard deviations above and below the mean RD values corresponds to red and green dotted lines, respectively. Strain H-3, containing a MIRU insertion relative to the inoculum strain, is represented by the black line. Shaded area corresponds to the MIRU coordinates. (**b**) Mean normalized mate pair distance values at MIRU locus 802 (+/− 100 bp). The color scheme is identical to plotted read depth values. Strain shown is again H-3.

After mapping to a single copy-number genome, RD and mate pair distance for each strain were mean normalized at each MIRU locus (+/− 100 bp) in our 16 strain panel. We classified strains which varied two standard deviations (SD) from the mean value for over 60% of a MIRU site as putative variants. To ensure this effect was limited to the MIRU locus, we assessed whether these strains also varied over two SD for more than 20% of the 100 basepair window surrounding the MIRU. Using these requirements, seven putative MIRU variants were identified, three by RD, three by PEM, and one by both approaches (Figure 1a, b, Table 1).

In order to assess the validity of the RD and PEM approaches we used Sanger sequencing to quantify the number of MIRU repeats at each of the 24 standard MIRU loci described previously [6]. To improve our estimate of the MIRU mutation rate, we assessed copy number in 17 additional strains isolated from cynomolgus macaques that were experimentally infected with the *Mtb* strain Erdman. All 33 strains were assessed at the 24-MIRU loci previously described. From the 792 loci sequenced, two of the four putative indels identified by PEM were confirmed, and no new variants were discovered (Table 1). Locus 802 in strain H-3 increased from three to four repeats and was identified by both RD and PEM, and locus 3192 in strain G-3 decreased from two to one copy and was identified solely by PEM.

**Figure 2:**
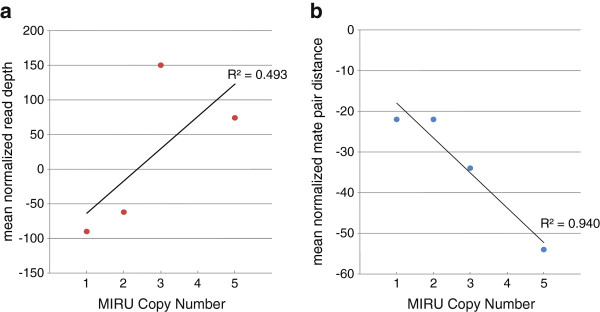
**Correlation between MIRU copy number and RD/mate pair.** (**a**) MIRU copy number vs. mean RD. The mean RD value for each locus was determined by averaging RD across the entire locus. All loci containing the same copy number as determined by Sanger sequencing were subsequently binned and averaged, providing a single RD value for each copy number observed in the data. (**b**) Graph of MIRU copy number vs. average mate pair distance. Average mate pair distance values were generated as described for RD.

With the number of repeats at each locus defined by Sanger sequencing, we determined the relationship between MIRU copy number and RD and PEM (Figure 2a, b, Table 1). Our results indicate a strong inverse correlation (r^2^ = .943) between MIRU copy number and mean normalized mate pair distance. Additionally, we find a positive correlation between mean normalized RD and MIRU copy number, though the correlation is weaker (r^2^ = .490). This is likely a reflection of the variance in read depth across the genome and between strains. This data suggests that PEM more closely correlates with MIRU copy number, consistent with more accurate detection of indels using PEM.

### Estimation of the MIRU mutation rate during the course of disease

**Figure 3:**
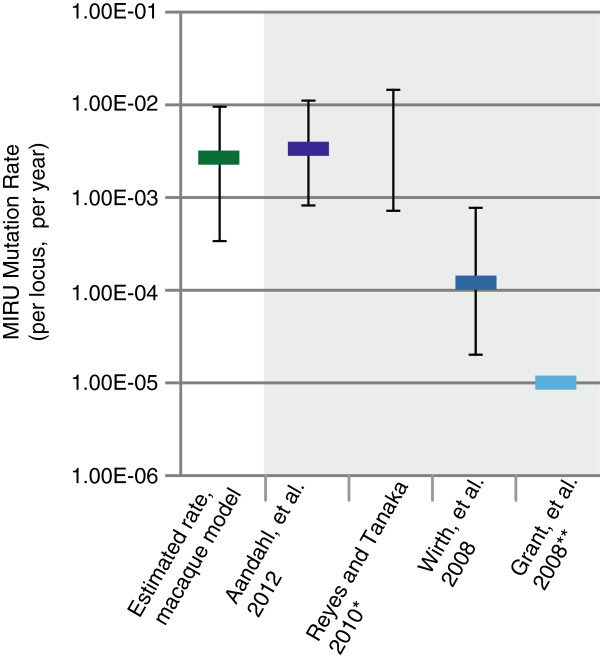
**Comparison of published MIRU mutation rates.** The per locus, per year published estimates of the MIRU mutation rate. Previous estimates shown in shaded grey. Error bars for all estimates shown represent 95% confidence intervals. *Reyes and Tanaka, 2010 provided a range of values but no mean value **Grant, et al. 2008 provided a single estimate and no confidence intervals.

We have estimated the per locus, per year, mutation rate by assessing MIRU CNV during the course of infection. The mutation rate, μ_MIRU,_ was calculated based on the number of MIRU indels identified by Sanger sequencing and the length of infection for each macaque, allowing us to estimate a per locus, per unit time rate. The rate was estimated by dividing the total observed CNVs by the total length of infection for each macaque, *t*_*(a-i)*_, the cumulative number of sequenced isolates per macaque, *g*_*(a-i)*_, and the cumulate number of MIRU loci sequenced, *l* (methods, equation 1). The MIRU mutation rate of our *in vivo* isolates was found to be 2.70 × 10^-03^ per locus, per year (95% CI: 3.30 × 10^-04^- 9.80 × 10^-03^). Our rate is most similar to the highest previous estimates derived from epidemiologic data, though the confidence interval overlaps with other estimates (Figure 3, Table 3).

**Table 3:** Values of published MIRU mutation rates

**Author**	**Mean MIRU mutation rate**	**Lower bound**	**Upper bound**
Estimated rate, macaque model	2.70 × 10^-3^	3.30 × 10^-4^	9.80 × 10^-3^
Aandahl et al. 2012	3.55 × 10^-3^	8.51 × 10^-4^	1.15 × 10^-2^
Reyes and Tanaka 2010	None	7.00 × 10^-4^	1.50 × 10^-2^
Wirth et al. 2008	1.23 × 10^-4^	1.96 × 10^-5^	7.93 × 10^-4^
Grant et al. 2008*	1.05 × 10^-5^	None	None

*Grant, et al. 2008 rate was converted from a per generation to a per year mutation rate.

## Discussion

Here we have utilized both conventional and next-generation sequencing approaches to define the number of copy variants at MIRU loci that arose during the course of macaque infection. Approaches to detect MIRU CNV by Illumina sequencing are increasingly important given the expanding use of WGS in molecular epidemiology. However, mapping ambiguities due to the repetitive nature of MIRU loci complicate copy number assessment by WGS. In this work, we sought to reduce mapping ambiguity and identify CNV in *Mtb* by mapping to a single copy-number genome. Sequencing technologies have advanced since the sequencing of these strains, and longer reads with less coverage variability may improve specificity in future work [31,32]. Additionally, it is thought that variability in both RD and PEM arises from local disparities in GC content [25,33]. Future work may exploit advances in correcting for GC bias [19,32,34-36], in combination with the approaches described here, to more reliably detect MIRU CNV from WGS data.

With the MIRU CNV identified in strains isolated from cynomolgus macaques, we have estimated the mutation rate at MIRU loci. Our *in vivo* MIRU mutation rate, 2.70 × 10^-03^ per locus, per year, is most consistent with the highest published estimates. Variability in these estimates may be partially driven by differences in MIRU loci analyzed, the epidemiology of the strains used, and differences in the assumptions of the models used to estimate a rate. The resulting differences in rate estimates have motivated extensive debate in the literature [15,37,38]. Our estimate is derived from an alternative, experimental approach, relying on the cynomolgus macaque model of TB infection to assess the mutation rate of the 24 loci standard used in MIRU typing. Though our analysis is somewhat limited by a relatively small signal, strikingly, our estimate closely aligns with previous rates.

What are the biologic consequences of a high mutation rate at MIRU loci? Interestingly, most MIRUs are located in intergenic regions and are hypothesized to be transcribed as part of a polycistronic operon. Several MIRU elements are located within the coding region of well-described two-component regulatory systems as well as genes essential for virulence and host adaptation [5]. It is interesting to hypothesize that rapid genetic variation at these MIRU loci may have effects on the transcription of the regulon, thus generating population diversity. Some evidence exists that CNV variation at MIRU loci may result in transcriptional changes of the downstream gene within a MIRU locus [39,40], though further characterization is required to establish the biologic relevance of these loci and the role of variation in MIRU elements.

Repeat variation is a well-established means of generating locus specific mutation in other microbial genomes [41,42]. The rate established here is comparable to tandem repeat variation rates in other organisms. For example, in *Bacillus anthracis* the estimated mutation rate of tandem repeat loci is reported to be roughly 10^-05^ to 10^-04^ per generation [43], while in pathogenic *E. coli* O157:H7, the rate is reported to be roughly 6.4 × 10^-04^ per generation [44]. The MIRU per generation mutation rate is between 5.6 × 10^-06^ and 7.64 × 10^-05^, using the lower (18 hours) and upper (240 hours) estimates of generation times previously described [17]. It is important to note the loci analyzed in this study were selected from the larger set of MIRU loci for their relative stability, making them ideal for typing. The rate of variation in the remaining loci may vary from the rate reported here, especially in loci previously identified as hypermutable [6]. While further work is needed to determine the biological consequences of MIRU variation, we have shown that there is potential for detecting MIRU variants by WGS and that the rate uncovered from macaque infection is consistent with the highest previous estimates.

## Conclusions

CNV in typing markers is an essential tool to differentiate and classify clinical strains, and quantitation of marker variation allows for enhanced interpretation of epidemiological data. In this study, we have used the macaque model of *Mtb* infection to estimate the MIRU mutation rate during the course of disease, and we have explored the use of WGS to assess MIRU copy number. Subsequent Sanger resequencing confirmed two of the four MIRU indels identified by PEM to a reduced copy genome, and from this we have estimated a per locus, per year mutation rate of 2.70 × 10^-03^. This value agrees with the higher published estimations of MIRU mutation rates. Further assessment of RD and PEM as indicators for copy number may streamline minisatellite detection via WGS.

## Methods

### Preparation of isolates and Illumina sequencing

Infection of macaques and isolation of strains was performed as previously described [45]. Briefly, cynomolgus macaques were infected with a low dose (roughly 25 CFU/macaque), virulent strain (*Mtb* Erdman) by bronchoscopy. Infected macaques were allowed to progress to either latent or active disease. Nine infected macaques were selected (four active, three latent, two reactivated) and 33 bacterial isolates from 17 different lesions were chosen for study. Colonies were expanded for extraction of genomic DNA as described previously. Minimal expansion occurred between strain isolation and genomic extraction. An Illumina Genome Analyzer (Illumina) was used for WGS of isolated strains. A detailed protocol of WGS data analysis has been previously published [17].

### WGS analysis of MIRU regions

75 basepair paired-end read data gathered from sequencing at the Broad Institute of MIT and Harvard were analyzed for MIRU CNV. Only strains containing sufficient sequencing coverage at the MIRU locus were analyzed. Sequences were mapped to a single copy H37Rv reference genome. This genome was created by reducing MIRU elements to a single copy at the 24 loci analyzed in the H37Rv genome [GenBank: AL123456]. If two MIRUs at the same locus contained a greater than 4 SNP difference, they were treated as unique MIRUs (denoted A and B) and were not collapsed to a single unit. Illumina fastq files were mapped to this reference genome with SSAHA2 using the Solexa defaults and allowing for paired end reads up to 700 bp [46]. Proper pairs were extracted using samtools. RD was calculated by including mate pairs traversing each reference coordinate with a perl script [47]. The two-sided Wilcoxon rank sum test was used to compare the difference in mate pair distances when mapping to the H37Rv genome versus the single copy-number genome (Mathworks, Natick MA). This analysis was done both on reads mapping within the MIRU and reads mapping in the surrounding +/−100 bp. For this comparison Illumina fastq files were mapped to the H37Rv genome using the same parameters as used when mapping to the single copy-number genome.

To identify CNV in MIRUs, RD and mate pair distance values were obtained for 16 strains at 24 MIRU loci and 100 basepairs upstream and downstream of the MIRU element. Values at each coordinate were normalized to its mean RD and mate pair distance values for the window assessed. MATLAB was used to generate plots, mean RD values and standard deviations (MathWorks, Natick MA).

### Sanger sequencing of isolates and estimation of MIRU mutation rate

33 strains were PCR amplified at the 24-MIRU locus set. Primers sequences for these were previously published [6]. Amplification was performed with the following reagents- 5 μl PCR buffer, 2% DMSO, 3 μl of 2.5 mM dNTPs, 3 μl of 10 mM of each primer, 20 ng of template, .5 μl of 250 U Taq polymerase, and water up to 50 μl. Thermocycler conditions were as follows: 95°C for 10:00, 30 cycles of: 95°C for :45, 68°C for :30, 72°C for :30, 72°C for 10:00. The MIRU mutation rate (μ_MIRU_) was estimated from the number of indel events observed by Sanger sequencing (Genewiz, Cambridge MA).

Equation 1 describes the estimation of the MIRU mutation rate:

(1) $${µ}_{\text{MIRU}}=n\;\text{indels}/\left(\sum \left(t_{\left(a-i\right)}\times g_{\left(a-i\right)}\times l\right)\right)$$

Where *n* indels = total number of MIRU insertion or deletion events, *t*_*(a-i)*_ = duration of infection per macaque (in days), *g*_*(a-i)*_ = number of strains analyzed per macaque, and *l* = total number of MIRU loci analyzed. *n* indels is divided by the sum of the product of *t, g*_*(a-i)*_, and *l*_(a-i)_ per macaque. Because infection length and isolates acquired is variable between macaques, the formula must sum the product acquired for each individual macaque. A Poisson distribution was used to model the number of indels and estimate the 95% confidence interval. Estimation of the mutation rate and the confidence interval was generated using MATLAB (MathWorks, Natick MA).

## Abbreviations

MIRU: Mycobacterial interspersed repetitive unit; Mtb: *Mycobacterium tuberculosis*; CNV: Copy number variation; WGS: Whole-genome sequencing; RD: Read depth; PEM: Paired-end mapping; TB: Tuberculosis; VNTR: Variable number of tandem repeats; SNP: Single nucleotide polymorphism; Indel: Insertion/deletion; SD: Standard deviation.

## Competing interests

The authors declare that they have no competing interests.

## Authors’ contributions

MNR and CBF designed and performed molecular studies, conducted the data analyses, prepared the figures and drafted the manuscript. MRC analyzed sequence data. PLL and JLF conducted the infection of the cynomolgus macaques, determined clinical state and acquired bacterial strains on necropsy. SMF designed the study, supervised experimental and molecular studies, and drafted the manuscript. All authors edited the manuscript.

## Supplementary Material

Additional file 1: Figure 1 Comparison of mate pair distance distribution from reads mapped to H37Rv versus single copy MIRU genome. (a) The distribution of mate pair distances from each sequencing read spanning the 3192 MIRU locus (+/− 100 bp) for strain G-2, for reads mapped to the H37Rv genome. (b) The distribution of mate pair distances from each sequencing read (for same strain and locus as (a)), for reads mapped to the single copy MIRU genome. For both (a) and (b), the bin size is set to 100. Bars in blue represent all MIRU sequencing reads +/− 100 bp while bars overlaid in green represent only the MIRU sequencing reads. Click here for file
