# Supplementary figures and images for: The mutation rate of mycobacterial repetitive unit loci in strains of M. tuberculosis from cynomolgus macaque infection
Source: BMC Genomics. 2013 Mar 5;14:145. doi: 10.1186/1471-2164-14-145 (PMC3635867; doi:10.1186/1471-2164-14-145)

Supplemental Figure 1

a)

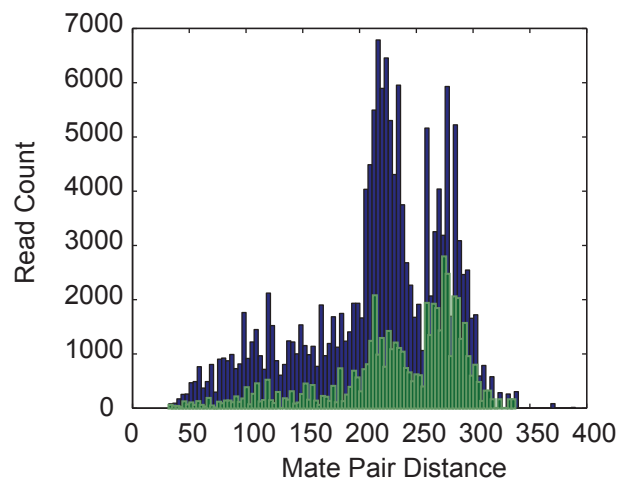

b)

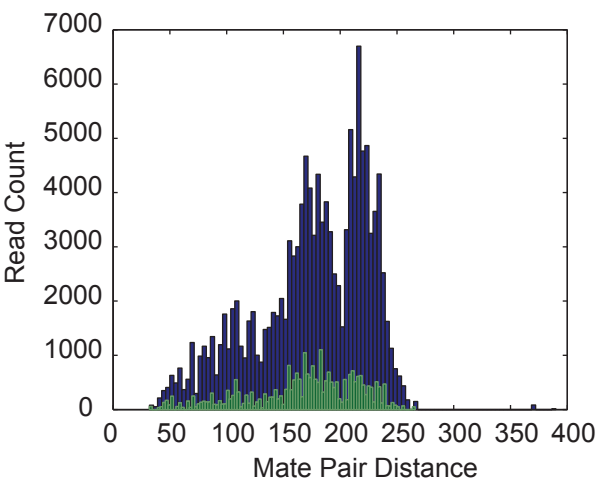

Supplement: Additional file 1: Figure 1 — Comparison of mate pair distance distribution from reads mapped to H37Rv versus single copy MIRU genome. (a) The distribution of mate pair distances from each sequencing read spanning the 3192 MIRU locus (+/− 100 bp) for strain G-2, for reads mapped to the H37Rv genome. (b) The distribution of mate pair distances from each sequencing read (for same strain and locus as (a)), for reads mapped to the single copy MIRU genome. For both (a) and (b), the bin size is set to 100. Bars in blue represent all MIRU sequencing reads +/− 100 bp while bars overlaid in green represent only the MIRU sequencing reads. [file 1471-2164-14-145-S1.pdf]
